# Response of the Endogenous Antioxidant Defense System Induced in RAW 264.7 Macrophages upon Exposure to Dextran-Coated Iron Oxide Nanoparticles

**DOI:** 10.3390/pharmaceutics15020552

**Published:** 2023-02-07

**Authors:** Mihaela Balas, Simona Liliana Iconaru, Anca Dinischiotu, Nicolas Buton, Daniela Predoi

**Affiliations:** 1 Department of Biochemistry and Molecular Biology, Faculty of Biology, University of Bucharest, 91-95 Splaiul Independentei, 050095 Bucharest, Romania; 2 HORIBA Jobin Yvon S.A.S., 6-18, Rue du Canal, CEDEX, 91165 Longjumeau, France; 3 National Institute of Materials Physics, 405A Atomistilor Street, 077125 Magurele, Romania

**Keywords:** iron oxide nanoparticles, dextran, RAW 264.7 macrophages, oxidative stress, antioxidant enzyme activity

## Abstract

Presently, iron oxide nanoparticles are the only ones approved for clinical use as contrast agents in magnetic resonance imaging (MRI). Even though there is a high demand for these types of nanoparticles both for clinical use as well as for research, there are difficulties in obtaining stable nanoparticles with reproducible properties. In this context, in this study, we report the obtaining by an adapted coprecipitation method of dextran-coated maghemite nanoparticles (ɤ-Fe_2_O_3_ NPs). The morphology and structure of the dextran-coated maghemite nanoparticles (ɤ-Fe_2_O_3_ NPs) were determined using scanning electron microscopy (SEM) and transmission electron microscopy (TEM). The TEM and SEM micrographs highlighted the obtaining of particles of nanometric size and spherical shape morphology. Furthermore, the high-resolution transmission electron microscopy (HRTEM), as well as selected area diffraction (SAED), revealed that the obtained samples presented the structure of cubic maghemite. In this study, we also explored the effects of the co-precipitation synthesized dextran-coated maghemite nanoparticles (ɤ-Fe_2_O_3_ NPs) on the redox status of macrophages. For cytotoxicity evaluation of these NPs, murine macrophages (RAW 264.7 cell line) were exposed to different concentrations of dextran-coated maghemite nanoparticles (ɤ-Fe_2_O_3_ NPs) corresponding to 0–500 μg Fe^3+^/mL and incubated for 24, 48, and 72 h. Intracellular iron uptake, changes in the oxidative stress parameters (reactive oxygen species production and malondialdehyde level), and the activity of antioxidant enzymes, as well as GSH concentration in cells, were evaluated after incubation with a lower (50 μg Fe^3+^/mL) and higher (500 μg Fe^3+^/mL) dose of NPs. The results indicated a significant decrease in RAW 264.7 cell viability after 72 h in the presence of NPs at concentrations above 25 μg Fe^3+^/mL. An important accumulation of NPs, dependent on dose and exposure time, was detected in macrophages, but it induced only a limited raise in the oxidative status. We showed here that the antioxidant capacity of RAW 264.7 macrophages was efficient in counteracting dextran-coated maghemite nanoparticles (ɤ-Fe_2_O_3_ NPs) toxicity even at higher doses.

## 1. Introduction

In the last decades, iron oxide nanoparticles (IONPs) have been extensively researched and used in the biomedical field as contrast agents, as carriers of biomolecules for drug delivery to specific organs and tissues, for gene therapy or as iron supplements for the treatment of patients suffering from anemia [1,2,3,4,5]. Over the years, researchers have made considerable efforts toward the elaboration of IONPs with improved physicochemical and surface properties [6,7,8,9,10]. In order to be considered for use in biomedical applications, the IONPs need to meet a series of criteria and, most of all, to be able to have excellent biocompatibility properties and to exhibit good biodistribution and pharmacokinetics properties [11,12,13]. It has been reported that the pharmacokinetic and biodistribution behavior of the IONPs is strongly correlated with the surface properties and physicochemical properties of the nanoparticles [14]. In this context, many attempts were made in order to improve the surface chemistry of IONPs and usually, those attempts involved the coating and/or functionalization of IONPs with different polymers, such as dextran, chitosan, starch and dextrin [3,15,16]. Amongst these, some have been successfully transferred to clinical trials [17], and some have even been approved for use by the U.S. Food and Drug Administration (FDA) [18] as contrast agents for MRI. One of the most successful polymers used in the functionalization of IONPs has been dextran, mainly due to its own excellent biological properties [19]. Dextran is used in the medical field as an antithrombotic because it has the ability to reduce blood viscosity [20]. Therefore, in this context, dextran-coated magnetic nanoparticles have been intensively studied over the years for their potential biomedical applications [21,22]. Even though there have been drawbacks with the iron oxide nanoparticles developed and approved for use over the years, there are still continuous worldwide efforts made for the optimizations of a synthesis route that will allow the obtaining of IONPs with reproducible properties and enhanced biological effects for biomedical applications. Therefore, in the last years, due to the tremendous efforts made by the scientific community, some iron oxide nanoparticles were approved by the FDA for clinical use, Feraheme^®^ was approved in the treatment of iron deficiency, and Combidex^®^ (U.S.) and Sinerem^®^ (Europe) were also approved to be used as magnetic resonance imaging (MRI) agents. In addition, Nanotherm^®^ (MagForce) was approved for being used in cancer treatment, and Lumirem^®^ was given approval to be used as an imaging agent for the oral gastrointestinal tract [23,24,25,26,27]. The biological effects of IONPs are dependent on various factors, including size, surface characteristics, dose, solubility, and cell type [28,29,30]. The interaction of NPs with biological components (e.g., serum proteins) is another important factor that modulates their bioreactivity and toxic effects on cells [31].

Upon intravenous administration, IONPs are opsonized (adsorption of plasma proteins on the NP′s surface) in the bloodstream before being phagocytized by macrophages and accumulated in the reticuloendothelial (RES) organs. This process promotes the accumulation of NPs in tissues, especially in tumor ones, due to the enhanced permeability and retention (EPR) effect [32]. Despite coating with plasma proteins, NPs may be cleared through opsonin-independent mechanisms by macrophages residing in the liver and spleen. Previously it was shown that either dextran or exposed IONPs could be directly recognized by macrophages [33]. Multiple alterations caused by exposure of macrophages to IONPs were previously reported [34,35]; however, the specific mechanisms of protective cellular response induced in the presence of various IONPs in macrophages are not clear.

The effects induced by these NPs on macrophage activation are a concern in terms of nanomaterial imaging, therapeutic efficacy, or systemic nanotoxicity. Excessive alterations drive macrophages to cell death, which has been associated with the development of several inflammatory diseases. Macrophages are equipped with a complex network of protective mechanisms against oxidative bursts (rapid release of ROS against pathogens) to survive during the inflammatory process [36]. How these mechanisms help macrophages to fight against oxidative stress induced by IONPs is an intriguing matter to reveal.

A deeper understanding of the interactions between NPs and macrophages is of great importance for researchers and clinicians as it can be directed toward generating biocompatible nanomaterials with specific surface characteristics fitting the biological environment.

In this context, we aimed to characterize the properties of newly synthesized dextran-coated maghemite nanoparticles (ɤ-Fe_2_O_3_ NPs) and to study the biological implications of their interaction with macrophages. Our focus was on cellular redox modulation after exposure to NPs, including oxidative stress induction and the activity of the endogenous antioxidant defense system. 

## 2. Materials and Methods

### 2.1. Sample Preparation

The dextran-coated maghemite nanoparticles (ɤ-Fe_2_O_3_ NPs) suspension was synthesized by the adapted coprecipitation method in agreement with anterior studies [37,38,39,40,41]. 

### 2.2. Physicochemical Characterization

Information regarding the morphology and structure of dextran-coated maghemite nanoparticles (ɤ-Fe_2_O_3_ NPs) were determined using transmission electron microscopy. The TEM studies were carried out using a CM20 (Philips FEI, Eindhoven, The Netherlands) transmission electron microscope (TEM) equipped with a Lab6 instrument.

Furthermore, the morphology of the dextran-coated maghemite nanoparticles (ɤ-Fe_2_O_3_ NPs) was investigated using the scanning electron microscopy technique with the aid of an FEI Quanta Inspect microscope. Furthermore, the SEM micrographs were used to determine the particle size distribution of the nanoparticles.

The mean hydrodynamic diameter and zeta potential of dextran-coated maghemite nanoparticles (ɤ-Fe_2_O_3_ NPs) suspension were determined by dynamic light scattering (DLS) using a ZetaSizer Nano ZS (Malvern Instruments Limited, UK). All measurements were effectuated 3 times at 25 °C.

The changes of residual mass of dextran, dextran-coated ɤ-Fe_2_O_3_ NPs and ɤ-Fe_2_O_3_ NPs with temperature were analyzed by TG analysis using a Perkin Elmer instrument. The thermogravimetric data were obtained from a thermogravimetric analyzer (TGA) performed under a dry nitrogen atmosphere from 30 to 1000 °C. The heating rate was 10 °C /min.

### 2.3. Biological Assays

#### 2.3.1. Cell Culture and Treatment

The macrophage RAW 264.7 cell line was purchased from American Type Culture Collection (ATCC, TIB-71, Manassas, VA, USA). The cells were grown in high-glucose Dulbecco Modified Eagle Medium (DMEM), supplemented with 10% fetal bovine serum (10270-106, origin South America, Gibco, Life Technologies, Carlsbad, CA, USA) and 1% antibiotic-antimycotic solution (A5955, Sigma, St. Louis, MO, US, USA) and maintained at 37 °C in a humidified atmosphere (95%) with 5% CO_2_. For treatment, the cells were seeded at a density of 5 × 10^4^ cells/mL and allowed to adhere for 24 h. Then, cells were exposed to different concentrations of dextran-coated ɤ-Fe_2_O_3_ NPs corresponding to 5, 10, 25, 50, 100, 250, and 500 μg Fe^3+^/mL and incubated for 24, 48, and 72 h. Untreated RAW 264.7 cells were used as control.

#### 2.3.2. MTT Assay

The cell viability was assessed using the colorimetric MTT (3-(4,5-dimethylthiazol-2-yl)-2,5-diphenyltetrazolium bromide) test relying on the reduction of the yellow MTT tetrazolium salt to purple formazan crystals in metabolically active cells. RAW 264.7 cells were seeded in a 24-well plate at a density of 5 × 10^4^ cells/mL/well. After treatment, the medium from each well was removed, and a volume of 250 μL 1 mg/mL MTT solution was added. After 2 h of incubation, the formazan crystals were dissolved in 250 μL 2-propanol solution. The absorbance was measured at 595 nm using a Tecan multi-plate reader (TecanGENios, Grödic, Germany). 

#### 2.3.3. Quantification of Intracellular Iron Content

The level of intracellular iron was measured as previously described [42]. After treatment with NPs, cells were trypsinized, washed with phosphate-buffered saline (PBS), centrifuged and counted. The cell pellet was digested in 5 N HCl for 24 h at 37 °C and then centrifuged at 2000× *g* rpm for 10 min. A volume of 50 μL of supernatant was mixed with 50 μL 1% ammonium persulfate solution (to convert the Fe^2+^ to Fe^3+^ ions) and 100 μL of 0.5 M potassium thiocyanate and shaken for 5 min in order to develop the red colored iron-thiocyanate. The formed complexes were spectrophotometrically detected at 450 nm. A standard curve with FeCl_3_•6H_2_O ranging from 0–250 μg Fe^3+^/mL was used to quantify the intracellular iron. The total amount of iron was related to the cell number.

#### 2.3.4. Measurement of ROS Production

The level of reactive oxygen species (ROS) generated in RAW 264.7 cells was estimated using the dye 2′,7′-dichlorodihydrofluorescein diacetate (H_2_DCF-DA; D6883, Sigma-Aldrich, St. Louis, MO, USA). Only two NPs concentrations, selected after cell viability evaluation, were investigated: 50 and 500 µg/mL. After treatment with NPs, the cells seeded in a 6-well plate were washed with PBS and incubated at 37 °C in the dark with a volume of 1.5 mL of 10 μM H_2_DCFDA solution for 30 min. Then, the cells were trypsinized, centrifuged and resuspended in 2 mL of PBS solution. The fluorescence of dichlorofluorescein (DCF) was spectrofluorimetrically recorded at 488 nm ex./515 nm em wavelength. The RFU values were related to the cell number in each sample.

#### 2.3.5. Preparation of Cell Lysate

RAW 264.7 cells were seeded in 75 cm^2^ culture flasks at a density of 2 × 10^6^ cells/flask. After treatment with NPs, the cell suspensions were centrifuged for 5 min at 1500× *g* rpm, and the cellular pellets were washed and resuspended in PBS. Cell lysis was obtained through ultrasonication 3 times for 30 s each, on ice, using a UP50H ultrasonicator (80% amplitude, 1 cycle) from Hielscher Ultrasound Technology (Teltow, Germany). The total extract was centrifuged at 3000× *g* rpm for 10 min at 4 °C, and the supernatants were collected. The protein concentration in each sample was measured using the Bradford method and a calibration curve of bovine serum albumin (BSA) ranging from 0 to 1.5 mg/mL protein.

#### 2.3.6. Detection of Lipid Peroxidation Products

The level of malondialdehyde (MDA) in RAW 264.7 cells was assessed as an indicator of lipid peroxidation via the thiobarbituric acid reaction, according to Dinischiotu et al. [43]. Briefly, a volume of 200 μL from cell lysate was mixed with 700 μL 0.1 N HCl and incubated for 20 min at room temperature. After adding 900 μL of 0.025 M thiobarbituric acid (TBA), the samples were incubated for another 65 min at 37 °C. Finally, a volume of 400 μL of PBS was added. The fluorescence of MDA (RFU) was recorded using a 520 nm/549 nm (excitation/emission) filter. A calibration curve with 1,1,3,3-tetramethoxy propane in the range of 0.05–5 μM was used to calculate the MDA concentration in each sample. The results are expressed as nmoles of MDA/mg protein.

#### 2.3.7. Quantification of Reduced Glutathione (GSH) Concentration

The intracellular GSH content was estimated in RAW 264.7 cells using the Glutathione Assay Kit (CS0260, Sigma-Aldrich, St. Louis, MO, USA). Thus, cell lysates were precipitated with 5-sulfosalicylic acid (1:1) and centrifugated at 10,000× *g* rpm for 10 min at 4 °C. A volume of 10 μL from the supernatant was mixed with 150 μL Assay buffer containing 5,50-dithiobis(2-nitrobenzoic acid) and incubated for 10 min at room temperature. The 5-thio-2-nitrobenozoic acid (TNB) formed was measured spectrophotometrically at 405 nm. A calibration curve of GSH (3.125–50 μM) was similarly prepared. The GSH levels were expressed as nmoles of GSH/mg protein.

#### 2.3.8. Measurement of Antioxidant Enzymatic Activity

i. Superoxide dismutase (SOD, EC 1.15.1.1) activity was measured according to the method of Paoletti et al. [44], which is based on NADPH oxidation by the superoxide anions. Superoxide anions were generated from oxygen molecules by the addition of triethanolamine: diethanolamine (100 mM) buffer pH 7.4, containing MnCl_2_-EDTA (100 mM /50 mM) and 2-mercaptoethanol (100 mM) to each sample of cell lysate. After the addition of 7.5 mM NADPH solution, the decrease of absorbance was followed at 340 nm for 10 min at 37 °C. A sample-free control was run in parallel. SOD activity was calculated in terms of U/mg of protein, where one unit (U) of activity was defined as the amount of enzyme required to inhibit the rate of NADPH oxidation of the control by 50%.

ii. Catalase (CAT, EC 1.11.1.6) activity was assessed by monitoring the disappearance of H_2_O_2_ at 240 nm, according to the Aebi method [45]. The cell lysate was mixed with 0.059 M H_2_O_2_ and 0.1 M K_2_HPO_4_/KH_2_PO_4_ buffer, pH 7.1, and the reaction kinetics was recorded spectrophotometrically for 1 min. CAT activity was calculated in terms of U/mg protein, where 1 U was the amount of enzyme that catalyzed the conversion of one μmole H_2_O_2_ in 1 min.

iii. Glutathione peroxidase (GPX, EC 1.11.1.9) activity was assessed by the Beutler method [46] from cell lysate, using 7 mM tert-butyl hydroperoxide and 2 mM NADPH as substrates. The conversion of NADPH to NADP^+^ was followed by recording the changes in absorption intensity at 340 nm for 5 min. One unit was defined as the amount of enzyme that catalyzes the conversion of 1 μmole of NADPH per minute. The activity was calculated as U/mg protein, using a molar extinction coefficient (ε_NADPH_) of 6.22 × 10^3^ M^−1^cm^−1^. 

### 2.4. Statistical Analysis

Data were obtained from 3 independent experiments and expressed in terms of mean ± standard deviation. The results from each sample were represented on graphs as percentages from the control (100%). Statistical analysis was performed through GraphPad Prism version 8.0.2 (GraphPad Software, Inc., San Diego, CA, USA) using two-way ANOVA variance with the Geisser-Greenhouse correction followed by Sidak’s multiple comparison test. Values of * *p* < 0.05, ** *p* < 0.01, and *** *p* < 0.001 were considered statistically significant (each sample vs. control).

## 3. Results and Discussion

Nanoparticles in general, and maghemite nanoparticles in particular, due to their adaptability, represent a promising platform that could improve therapeutic delivery and controlled release in the area of different affected organs. The size of the particles and their stability are two very important parameters that characterize the behavior of nanoparticles. Thus, studies that contribute to the understanding of these nanometric suspensions are very important. 

**Figure 1:**
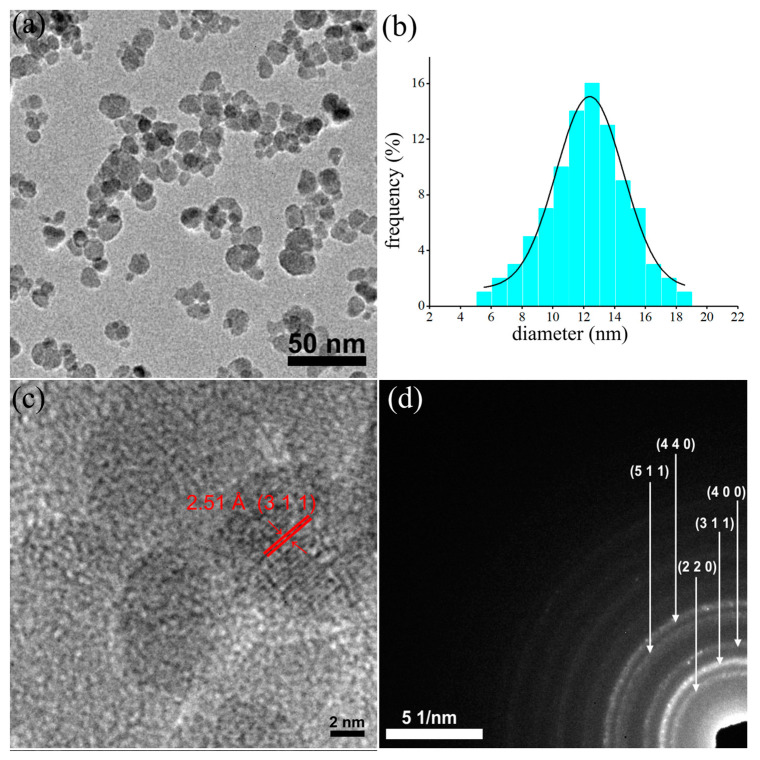
TEM image (**a**), particle size distributions obtained from large area TEM image (**b**), high-resolution TEM (HRTEM) image (**c**) and selected area electron diffraction (SAED) (**d**) of dextran-coated maghemite nanoparticles (ɤ-Fe_2_O_3_ NPs).

The morphology and structure of the prepared dextran-coated maghemite nanoparticles (ɤ-Fe_2_O_3_ NPs) were investigated by TEM (Figure 1a–d). The large bright field TEM image (Figure 1a) revealed that the dextran-coated iron oxide nanoparticles have a uniform particle distribution of nanometric sizes and spherical shapes. Moreover, the particle size distribution was also determined by measuring the mean diameter, D, of approximately 500 particles. 

The average grain size distribution obtained from the TEM image is depicted in Figure 1b, and it was emphasized that the particle size distribution was approximately 12.4 nm. Furthermore, the high-resolution TEM image, presented in Figure 1c, revealed the existence of regular fringes in the nanoparticle having a distance of 2.51 Å, which is characteristic of the (311) interplanar distance of the cubic maghemite. The clear lattice fringe observed in the HRTEM image highlighted the obtaining of nanoparticles with a crystalline nature. The SAED patterns obtained for the dextran-coated iron oxide nanoparticles were indexed to be of cubic maghemite, ɤ-Fe_2_O_3_, and the obtained rings were attributed to the (220), (311), (400), (422), (511) and (440) planes, respectively in agreement with existing reported data [47,48]. 

**Figure 2:**
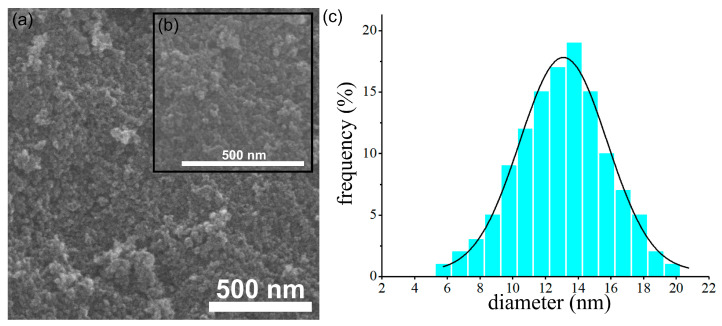
SEM image (**a**), inset of high magnification SEM image (**b**) and particle size distributions obtained from SEM image (**c**) of dextran-coated maghemite nanoparticles (ɤ-Fe_2_O_3_ NPs).

Furthermore, additional information about the morphology of the dextran-coated maghemite nanoparticles (ɤ-Fe_2_O_3_ NPs) was also gathered using scanning electron microscopy. The results obtained from the SEM studies are depicted in Figure 2a–c. The SEM micrograph presented in Figure 2a, as well as the inset of the high magnification SEM image (Figure 2b), confirmed the data obtained from the TEM studies and revealed the obtaining of particles having nanometric sizes and spherical shapes. In addition, the SEM micrograph also depicted the uniformity a homogeneity of the particles. More than that, the particle size distribution obtained using the SEM micrograph determined the grain size distribution at 13.1 nm, which is consistent with the one obtained from the TEM image. 

The stabilization of maghemite nanoparticles by the adsorption of a layer of dextran around the particles is very important in biological and medical applications. The formation of the dispersant layer (adlayer, in our case, dextran) was very important for the stabilization of maghemite suspensions. It is known that the thin layers on the surface of the particles lead to the agglomeration of the particles. For this purpose, DLS studies were carried out to determine the hydrodynamic diameter of maghemite nanoparticles covered with dextran in suspension. The zeta potential was also determined for the studied suspensions.

**Figure 3:**
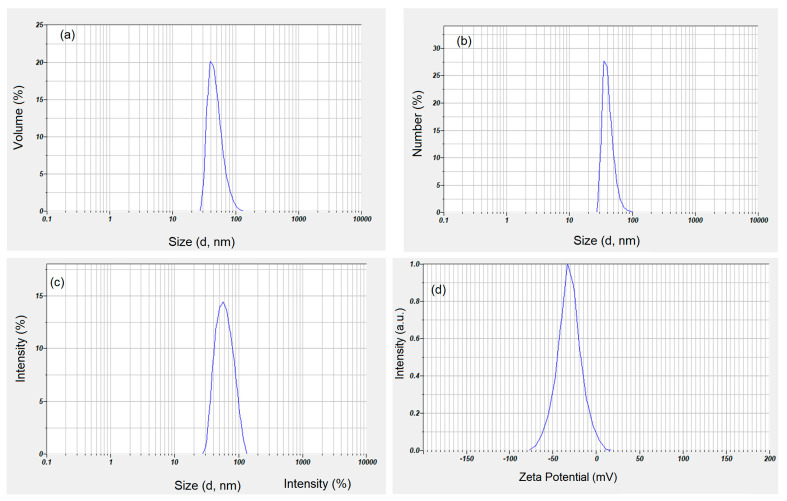
Volume size distribution (**a**), particle size by number distribution (**b**), intensity size distribution (**c**) and zeta potential (**d**) for maghemite nanoparticles coated with dextran in suspension.

Figure 3 shows the distribution based on volume (a) and the distribution based on numbers (b). Both the distribution based on volume and the one based on numbers clearly show that the suspension of maghemite nanoparticles covered with dextran does not present aggregates. As can be seen from the volume size distribution, the analyzed sample is composed of approximately 45.4 nm particles (Figure 3a). The number distribution presented in Figure 3b was monomodal, with an average peak at 38 nm. Figure 3c revealed the intensity particle size distribution obtained for maghemite nanoparticles coated with dextran in suspension. The graph shows a monomodal distribution with a distinct peak at 45.7 nm without indicating the presence of aggregates in the studied sample. The ratio D_H_/D_TEM_ representing the hydrodynamic diameter (D_H_) calculated from DLS studies and the nanoparticle diameter calculated by TEM (D_TEM_) was 3.7 nm. On the other hand, the ratio D_H_/D_SEM_ representing the hydrodynamic diameter (D_H_) calculated from DLS studies and the nanoparticle diameter calculated by SEM (D_SEM_) was 3.5 nm. Both the D_H_/D_TEM_ ratio and the D_H_/D_SEM_ ratio show that the dextran present on the surface of the maghemite nanoparticles results in an increase in the size of the obtained sample. This result is in full agreement with previous studies [49,50,51,52]. The dispersion stability of dextran-coated maghemite suspension was measured through the absolute value of zeta potential. According to previous studies [53], it can be said that a nanofluid has a colloidal dispersion stability if the absolute zeta potential is greater than 30 mV. The colloidal stability of dextran-coated maghemite suspension using zeta potential was presented in Figure 3d. The zeta potential measured for dextran-coated maghemite suspension was −38.4 mV. Since the measured value is higher than 30 mV, it can be said that the dextran-coated maghemite suspension is at good colloidal stability condition.

The numerical value of the polydispersity index (PDI) of the ɤ-Fe_2_O_3_ NPs was 0.165. In agreement with previous studies [54,55], the PDI is a representation of the population size distribution in a sample. Moreover, previous studies [56] have demonstrated that values lower than or equal to 0.2 are considered to be accepted in practice for nanoscale materials containing polymers. Moreover, PDI values between 0.0 and 0.5 highlight the fact that the analyzed sample is uniform in relation to the particle size [57]. The DLS and zeta potential studies showed that the dextran layer on the surface of the maghemite nanoparticles has a fairly significant thickness, which led to very good stability, as could be seen from the zeta potential analysis.

**Figure 4:**
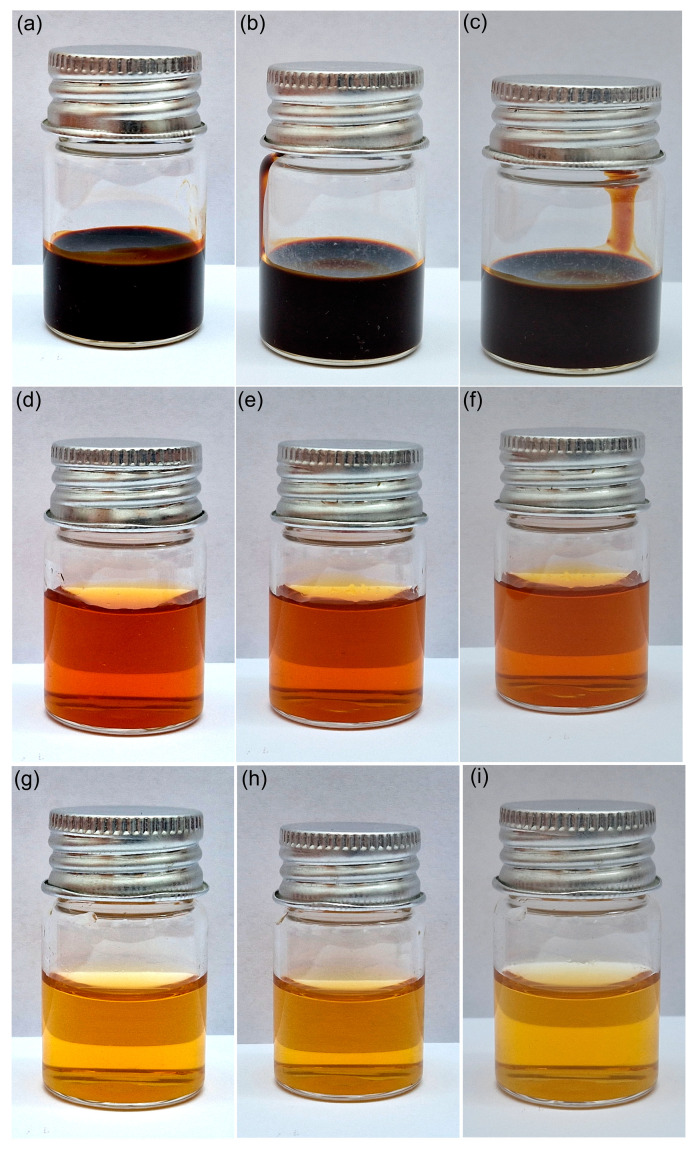
Visual appearance of prepared dextran-coated ɤ-Fe_2_O_3_ NPs (**a**–**c**), dextran-coated ɤ-Fe_2_O_3_ NPs with 500 µg Fe^3+^/mL (**d**–**f**) and dextran-coated ɤ-Fe_2_O_3_ NPs with 50 µg Fe^3+^/mL (**g**–**i**) observed after 1 day (**a**,**d**,**g**), 3 days (**b**,**e**,**h**) and 7 days (**c**,**f**,**i**).

Furthermore, the stability of the dextran-coated ɤ-Fe_2_O_3_ NPs was also investigated visually. Images of dextran-coated ɤ-Fe_2_O_3_ NPs suspensions kept at room temperature in glass vials over different periods of time are depicted in Figure 4a–i. The images were taken after 1 day, 3 days and 7 days. As can be observed from the images presented in Figure 4, the suspensions were stable over time, and there was no sign of precipitation or deposition of the nanoparticles at the bottom of the vials. Moreover, Figure 4a depicts the image of the dextran-coated ɤ-Fe_2_O_3_ NPs suspensions obtained from the synthesis without dilution. The images of the vials taken after 1 day, 3 days, and 7 days emphasized that the obtained suspension is stable and there were no signs of particle agglomeration or particle deposition in the investigated time interval. Furthermore, the diluted dextran-coated ɤ-Fe_2_O_3_ NPs suspensions used in the biological assays were also studied, and their images after 1 day, 3 days and 7 days are depicted in Figure 4d–i. The results were in agreement with previous studies and determined that the obtained suspensions were stable in time.

**Figure 5:**
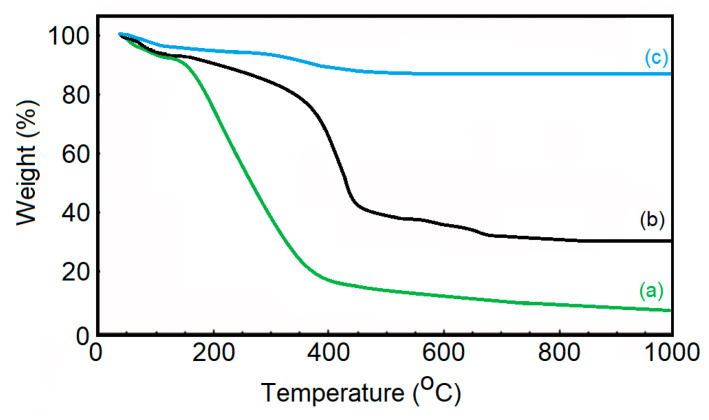
TGA curves of dextran (**a**), dextran-coated ɤ-Fe_2_O_3_ NPs (**b**) and ɤ-Fe_2_O_3_ NPs (**c**).

The TGA curves of dextran (a), dextran-coated ɤ-Fe_2_O_3_ NPs (b) and ɤ-Fe_2_O_3_ NPs (c) in the temperature range of 30−1000 °C are presented in Figure 5. For all the samples, we observed an initial small weight loss (between 30–150 °C). This small weight loss was due to the amount of water physically absorbed from their surface and the dehydroxylation of hydroxyl groups on the surface. This behavior was in accordance with the other previous results [58,59]. As can be seen in Figure 5, the decomposition of dextran takes place in a single step in the temperature range of 180–750 °C. The thermal decomposition of ɤ-Fe_2_O_3_ NPs takes place in two stages. The first stage of dextran degradation takes place with a mass loss in the range of 150–400 °C. The second stage of dextran degradation takes place in the range of 400–700 °C. After 700 °C, we have no more mass loss, which shows that the dextran has been completely decomposed, and what remains are only maghemite nanoparticles.

The obtained results were in good agreement with previous studies [60,61,62]. According to previous studies [63], the percentage of dextran molecules attached to the surface of ɤ-Fe_2_O_3_ NPs can be simply calculated by measuring the difference in residual weight between uncoated ɤ-Fe_2_O_3_ NPs and dextran-coated ɤ-Fe_2_O_3_ NPs. This result reveals that the coating efficiency of the ɤ-Fe_2_O_3_ NPs was about 52.13 %. The TGA results are in agreement with the results obtained from DLS and zeta potential studies. Moreover, our results obtained from TGA analysis are in agreement with previous studies [63] that obtained a dextran-coating efficiency of magnetite nanoparticles of approximately 50.12% and 53.89%, respectively.

**Figure 6:**
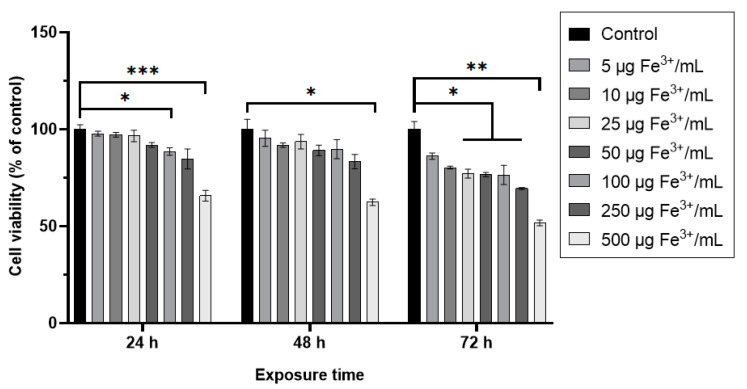
Cell viability of RAW 264.7 cells after 24, 48, and 72 h exposure to different doses of dextran-coated ɤ-Fe_2_O_3_ NPs (corresponding to 5–500 μg Fe^3+^/mL). Data (*n* = 3) are expressed as relative values (%) related to control ± SD. The results were considered statistically significant at * *p* < 0.05, ** *p* < 0.01 and *** *p* < 0.001 (sample vs. control).

The cell viability of RAW 264.7 murine cells exposed to dextran-coated maghemite nanoparticles (ɤ-Fe_2_O_3_ NPs) in a concentration range of 5–500 μg Fe^3+^/mL varied in a dose and time-dependent manner (Figure 6). For doses below 250 μg Fe^3+^/mL, no significant decrease of viability, compared to control, was noticed after 24 and 48 h from treatment except for the dose of 100 μg Fe^3+^/mL at 24 h. For the same exposure intervals, the dose of 500 μg Fe^3+^/mL induced a significant reduction of cellular viability by 35% and 38%, respectively, compared to control cells. After 72 h of treatment, all doses above 25 μg Fe^3+^/mL caused a significant decrease in this parameter. Two doses of NPs of 50 and 500 μg Fe^3+^/mL have been chosen for the following determinations.

**Figure 7:**
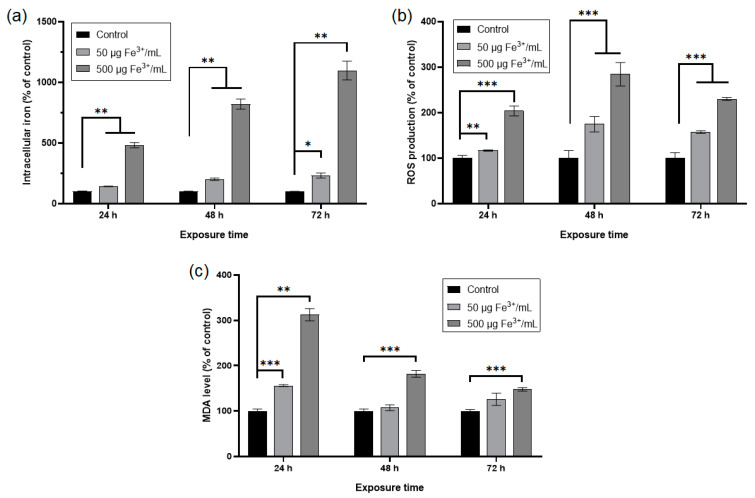
Cellular iron uptake and levels of oxidative stress parameters in RAW 264.7 cells after 24, 48, and 72 h exposure to two doses of dextran-coated ɤ-Fe_2_O_3_ NPs corresponding to 50 and 500 μg Fe^3+^/mL. (**a**) intracellular iron content quantified by iron-thiocyanate reaction; (**b**) level of reactive oxygen species detected by H_2_DCF-DA assay; (**c**) level of malondialdehyde estimated by TBA reaction. Data (*n* = 3) are expressed as relative values (%) related to control ± SD. The results were considered statistically significant at * *p* < 0.05, ** *p* < 0.01 and *** *p* < 0.001 (sample vs. control).

The internalization of NPs was quantified by measuring the level of iron in the treated RAW 264.7 cells with NPs corresponding to doses of 50 μg Fe^3+^/mL and 500 μg Fe^3+^/mL, respectively (Figure 7a). The intracellular concentration of Fe^3+^ increased in time, depending on the applied dose. The lower dose determined a significant increase of Fe^3+^ concentration by about 42%, 101%, and 132% after 24 h, 48 h, and 72 h, respectively, whereas the higher one generated a significant raise of the intracellular Fe^3+^ level by about 383%, 721%, and 997% respectively, compared to control cells. According to Figure 7b, the production of ROS in RAW 264.7 murine cells exposed to NPs increased in a concentration and time-dependent manner up to 48 h of exposure, followed by a slight decrease after 72 h. The dose of 50 μg/mL determined a significant raise of ROS levels by about 17%, 75%, and 57% after 24 h, 48 h, and 72 h, respectively, whereas the higher dose generated a significant increase of these by 104%, 185% and 130% after the same intervals of exposure, compared to control cells. Malondialdehyde (MDA) is an end-product of the oxidative degradation of polyunsaturated fatty acids (PUFA) and is an indicator of cellular oxidative stress. In the RAW 264.7 murine cells exposed for 24 h to both doses of NPs, the MDA concentrations increased significantly by 56% (lower dose) and 212% (higher dose) in comparison with control cells (Figure 7c). The treatment of RAW 264.7 cells with a dose of 50 μg Fe^3+^/mL for 48 h and 72 h generated insignificant increases in MDA levels by 7% and 25%, respectively, compared to the control. Instead, exposure to NPs corresponding to 500 μg Fe^3+^/mL determined significant raises of MDA concentrations by 82% and 48% after 48 h and 72 h, respectively, relative to the control.

**Figure 8:**
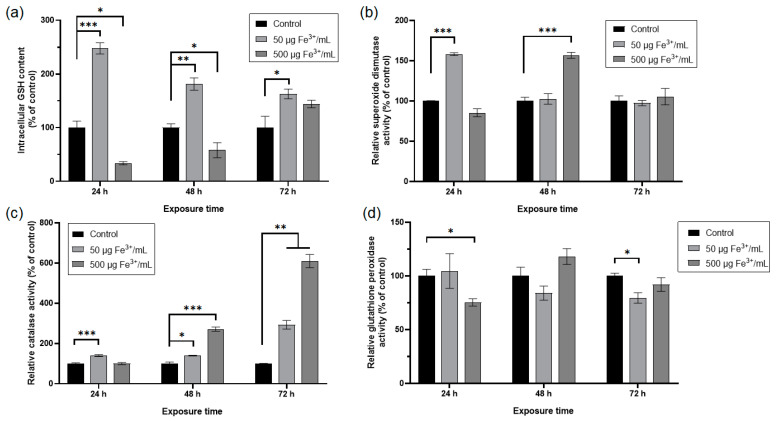
Antioxidant response of RAW 264.7 cells after 24, 48, and 72 h exposure to two doses of dextran-coated ɤ-Fe_2_O_3_ NPs corresponding to 50 and 500 μg Fe^3+^/mL. (**a**) level of intracellular GSH concentration; (**b**) enzymatic activity of SOD; (**c**) enzymatic activity of CAT; (**d**) enzymatic activity of GPX. Data (*n* = 3) are expressed as relative values (%) related to control ± SD. The results were considered statistically significant at * *p* < 0.05, ** *p* < 0.01 and *** *p* < 0.001 (sample vs. control).

The cellular antioxidant defense system is formed by non-enzymatic and enzymatic molecules. Reduced glutathione (γ-glutamyl-cysteinyl-glycine) is a tripeptide that represents the main non-enzymatic molecule. The exposure of RAW 264.7 cells to NPs corresponding to 50 μg Fe^3+^/mL determined a significant increase of GSH level by 148% after 24 h related to the control (Figure 8a). At longer periods of treatment, this increase was by about 81% and 63% after 48 h and 72 h, respectively, compared to the control. Even if this increase is diminished in a time-dependent manner, the level of this non-enzymatic antioxidant remains higher in comparison with the control. In the case of treatment with the higher dose, the level of GSH decreased by 66% and 42% after 24 h and 48 h, respectively, and increased by 44% after 72 h, although at this time point, the result was not statistically significant.

Antioxidant enzymes such as superoxide dismutase (SOD), catalase (CAT), and glutathione peroxidase (GPX) work together to counteract the toxic ROS produced in cells. 

The specific activity of SOD (Figure 8b) increased in the cells exposed to NPs corresponding to 50 μg Fe^3+^/mL by 58% after 24 h and returned to the control level after 48 h and 72 h. However, post-treatment with NPs corresponding to 500 μg Fe^3+^/mL, this enzymatic activity decreased by 15% after 24 h, increased significantly by 57% after 48 h and returned to the control level after 72 h.

The specific activity of CAT (Figure 8c) increased significantly by about 40% after 24 h and 48 h of exposure to NPs corresponding to 50 μg Fe^3+^/mL. After 72 h, this enzymatic activity raised significantly by 193% compared to the control. The treatment of RAW 264.7 cells with NPs corresponding to 500 μg Fe^3+^ /mL determined no significant change in the specific activity of CAT after 24 h and a significant increase by 171% and 510%, respectively, after 48 h and 72 h. 

The specific activity of GPX (Figure 8d) in the RAW 264.7 cells exposed to NPs corresponding to 50 μg Fe^3+^/mL decreased significantly only after 72 h, by 20% related to control. High dose administration significantly inhibited the specific activity of GPX by 25% only after 24 h, then after 48 h, only a slight activation by 18% was noticed, and after 72 h, the level returned to normal.

The RAW 246.7 murine macrophage cell line is used to screen natural and engineered products to predict their potential effect in vivo [64]. Macrophage responses to different NPs are influenced by dose, size, composition, and surface properties and uptake these by endocytosis or phagocytosis, undergoing polarization [65]. 

In the presence of serum-containing culture media, NPs’ surfaces form protein coronae that confer properties involved in their interaction with cells. Previously, it was shown that the uptake of NPs by macrophages was directly related to the amounts of proteins adsorbed on the NPs’ surface [66]. Also, studies in vivo revealed that dextran-coated supermagnetic iron (SPIO) NPs presented on their surface, mannan-binding lectins bound to the dextran coating, and histidine-rich glycoprotein and kininogen bound to the iron oxide part. Also, secondary binders such as complement lectin and contact clotting factors were identified [33]. 

In our study, the uptake of NPs containing protein corona is highlighted by the increase of Fe^3+^ ions concentration in the treated RAW 246.7 cells, these ions being released from the maghemite core. There is a significant probability that Fe^3+^ ions to be complexed by low molecular weight compounds such as citrate, nucleotides, and glycosaminoglycans. These Fe^3+^-chelates could be reduced to Fe^2+^ by NAD(P)H-dependent flavoenzymes, lipoyl dehydrogenase being probably the dominant catalyst within cells [67]. As a result, the level of Fe^2+^ probably increased, and the Fenton reaction occurred, generating hydroxyl radicals (HO•) and hydroxide ions (OH−). 

On the other hand, due to the interaction of NPs with RAW 246.7 cells, NADPH oxidase could be activated, producing superoxide anions (O_2_^•−^), as was proved in the case of other types of NPs [68]. Metabolism of iron occurs predominately in mitochondria, and its overload can also affect mitochondrial function because this transition metal is involved in ROS production, which can diminish the expression of mitochondrial-encoded respiratory chain subunits [69]. Superoxide passage by simple diffusion inside cells is highly unfavorable [70], but this anion can use anion channels of membranes to enter cells [71]. Superoxide can also react with H_2_O_2_ in the Haber-Weiss reaction, catalyzed by iron [72], also generating hydroxyl radicals (HO•). 

ROS can attack PUFA and generate MDA, which is an end-product of lipid peroxidation. These considerations are supported by the same pattern of cellular Fe^3+^ and ROS levels up to 48 h of exposure. The highest level of MDA was registered after 24 h, being correlated with ROS concentration. Later on, MDA concentrations decreased in a time-dependent manner, probably due to the activation of the non-enzymatic and enzymatic antioxidant systems.

Alternatively, superoxide can be dismutated in the reaction catalyzed by SOD, generating H_2_O_2_ and molecular oxygen [73]. In mammalian cells, there are 2 types of SOD: Cu/Zn-SOD, present mainly in the cytosol, and mitochondrial MnSOD. The lower dose (50 μg Fe^3+^/mL) of NPs determined an increase in the total SOD activity after 24 h of exposure, followed by a decrease to the control level after 48 h and 72 h, respectively. Probably, this decrease occurred due to the significant level of H_2_O_2_ generated, which could inhibit Cu/Zn-SOD [74]. Also, the exposure to the higher dose (500 μg Fe^3+^/mL) of NPs slightly diminished the total SOD after 24 h, probably due to the same reason. Instead, the increase of total SOD after 24 h in cells exposed to the lower dose we assumed it might be a consequence of the Mn-SOD activation. As shown in a previous study [75], Mn-SOD activity could be induced in macrophages as a mechanism for the acquirement of oxidative stress resistance through the participation of PARP1 in the regulation of Mn-SOD gene expression. Mn-SOD is possible to respond to an endogenous redox imbalance that involves superoxide leakage as a result of disruption of mitochondria functions in iron-overloaded cells. However, how much Mn-SOD contributes to the strongly elevated level of total SOD after 24 h is unclear and needs to be further investigated. Possibly, after 48 h, the incorporation of iron instead of Mn into Mn-SOD switched its activity to a prooxidant peroxidase that utilizes H_2_O_2_ for the oxidation of other molecules [76]. Previously, it was also proved that the accumulation of iron decreased the expression and activity of Mn-SOD [77]. In our opinion, after 48 h, the high concentration of Fe^2+^ generated an increase in Mn-SOD activity due to the two-ion switch. After 72 h, possibly the accumulation of Fe^2+^ decreased the expression and activity of Mn-SOD.

CAT catalyzes the reaction of decomposition of H_2_O_2_, a product of the reaction catalyzed by SOD, in water and molecular oxygen. CAT is a porphyrin-containing tetramer that is located mainly in peroxisomes [78]. The variation of CAT-specific activity in time can be correlated with that of total SOD in the case of treatment with the lower dose. In the case of the higher dose, the significant increase of CAT-specific activity after 48 h could be correlated with the high total SOD activity. Possibly, the high quantity of H_2_O_2_ generated after the switch of Mn with iron ions was involved in the up-regulation of Mn-SOD and CAT gene transcription [79].

The level of H_2_O_2_ produced in cells can be controlled not only by CAT activity but also by GPX. This latter enzyme catalyzes H_2_O_2_ decomposition in the presence of GSH to form water and oxidized glutathione (GSSG). The Michaelis constant for H_2_O_2_ is 50 times higher for CAT compared to that of GPX [80]. GPX is the first enzyme that is activated under high levels of ROS, playing an important role as a first line of defense against oxidative stress [81]. However, taking into account the high level of CAT activity, we could consider that the quantity of H_2_O_2_ generated was removed by CAT action. 

GPX also catalyzes the reaction between GSH and lipid peroxides, playing a role in the detoxification of these molecules. After exposure to the lower dose, GPX-specific activity was not changed after 24 h and decreased after the other time intervals, suggesting that this enzyme was not involved in the removal of MDA. Taking into account the pattern of GSH variation corresponding to this dose, probably this tripeptide was a substrate for glutathione-S-transferase (GST), which could be implicated in MDA removal [82]. At the same time, in the case of treatment of RAW 246.7 cells with the higher dose of NPs, GPX was not efficient in MDA detoxification.

In the present study, at a lower dose (50 μg Fe^3+^/mL), the GSH level decreased after 24 h and increased in a time-dependent manner up to 72 h when it became higher than the control level. The higher dose (500 μg Fe^3+^/mL) increased the GSH level by 150% after 24 h exposure and decreased later on, remaining higher than the control level at all time intervals. Previous studies revealed that dextran-coated maghemite nanoparticles (ɤ-Fe_2_O_3_ NPs) modulate the GSH level in a cell-type-dependent manner [47,83].

Our results highlight the need to evaluate in-depth the interactions between IONPs and cells to take full advantage of the intrinsic properties of these NPs in biological systems.

## 4. Conclusions

The present study was focused on the response of the endogenous antioxidant defense system induced in RAW 264.7 macrophages upon exposure to dextran-coated maghemite nanoparticles (ɤ-Fe_2_O_3_ NPs). The dextran-coated maghemite nanoparticles (ɤ-Fe_2_O_3_ NPs) were obtained using an adapted coprecipitation method. The morphology of the dextran-coated maghemite nanoparticles (ɤ-Fe_2_O_3_ NPs) was investigated by TEM and SEM studies. The SEM and TEM micrographs highlighted that the particles were well-dispersed, presenting a nanometric size distribution and spherical shape. The particle size distributions determined from TEM and SEM micrographs were 12.4 nm and 13.1 nm. Moreover, the HRTEM studies revealed clear lattice fringes, which suggested a good crystallinity of the samples. In addition, the SAED patterns obtained for the dextran-coated iron oxide nanoparticles were indexed to be of cubic maghemite, ɤ-Fe_2_O_3_. As observed in the DLS studies, the dextran layer on the surface of the maghemite particles is large enough to ensure the very good stability of these nanoparticles. Therefore, maghemite nanoparticles functionalized with dextran could be successfully applied in different biological and medical fields.

RAW 246.7 cells efficiently counteracted the oxidative stress induced by the exposure to these NPs. This statement is supported by the increased GSH level and the diminished MDA content in the RAW 246.7 cells after the maximum exposure time.

The results obtained in this study emphasized that dextran-coated maghemite nanoparticles (ɤ-Fe_2_O_3_ NPs) obtained by an adapted coprecipitation method could be used in the medical and pharmaceutical fields due to their physicochemical and biological properties.

## Data Availability

Data is available on demand from the corresponding authors.
